# Tracking a Non-Cooperative Target Using Real-Time Stereovision-Based Control: An Experimental Study

**DOI:** 10.3390/s17040735

**Published:** 2017-03-31

**Authors:** Tomer Shtark, Pini Gurfil

**Affiliations:** Department of Aerospace Engineering, Technion—Israel Institute of Technology, Haifa 32000, Israel; tomershtark@gmail.com

**Keywords:** tracking, stereovision, real-time control

## Abstract

Tracking a non-cooperative target is a challenge, because in unfamiliar environments most targets are unknown and unspecified. Stereovision is suited to deal with this issue, because it allows to passively scan large areas and estimate the relative position, velocity and shape of objects. This research is an experimental effort aimed at developing, implementing and evaluating a real-time non-cooperative target tracking methods using stereovision measurements only. A computer-vision feature detection and matching algorithm was developed in order to identify and locate the target in the captured images. Three different filters were designed for estimating the relative position and velocity, and their performance was compared. A line-of-sight control algorithm was used for the purpose of keeping the target within the field-of-view. Extensive analytical and numerical investigations were conducted on the multi-view stereo projection equations and their solutions, which were used to initialize the different filters. This research shows, using an experimental and numerical evaluation, the benefits of using the unscented Kalman filter and the total least squares technique in the stereovision-based tracking problem. These findings offer a general and more accurate method for solving the static and dynamic stereovision triangulation problems and the concomitant line-of-sight control.

## 1. Introduction

### 1.1. Cooperative vs. Non-Cooperative Targets

An important problem in the field of computer vision is estimating the relative pose. Numerous past studies addressed this problem, dealing with objects on which some information is a priori available; these objects are referred to as *cooperative targets*.

To estimate the relative pose with respect to cooperative targets, Fasano, Grassi and Accardo [1] used light emitting diodes, while Woffinden and Geller [2] used known shapes as features, placed at known positions on the target. Terui, Kamimura and Nishida [3] used a known 3D model of the object, a model matching technique, and 3D feature points obtained from stereo matching.

The problem of estimating the position, velocity, attitude and structure of a non-cooperative target significantly surpasses the cooperative problem in complexity. Segal, Carmi and Gurfil [4,5] used two cameras for finding and matching a priori unknown feature points on a target. These feature points were fed to an Iterated Extended Kalman Filter (IEKF) [6], which was based on the dynamics of spacecraft relative motion.

Also, the target’s inertia tensor was estimated using a maximum a posteriori identification scheme. Lichter and Dubowsky [7,8] used several vision sensors, which were distributed fairly uniformly about the target, in order to estimate its properties. Sogo, Ishiguro and Trivedi [9] used multiple omnidirectional vision sensors and a background subtraction technique in order to measure the azimuth angle of the target from each vision sensor. Jigalin and Gurfil [10] compared the Unscented Kalman Filter (UKF) [11,12,13] and the IEKF, and studied the effects of increasing the number of cameras.

The problem of controlling robot motion using visual data as feedback, is commonly referred to as visual servoing. Cai, Huang, Zhang and Wang [14] presented a novel dynamic template matching that may be used for a monocular visual servoing scheme of a tethered space robot in real time using one camera. The proposed matching method detects specific regions on satellites with high accuracy. It also includes experiments for verifying the proposed matching method, showing promising results. Chen, Huang, Cai, Meng and Liu [15] proposed a novel non-cooperative target localization algorithm for identification of a satellite’s brackets, which is a proper grasping position for a tethered space robot. Reference [15] also includes experiments on standard natural images and bracket images, which demonstrated its robustness to changes in illumination. Huang, Chen, Zhang, Meng and Liu [16] proposed a control method of visual servoing based on the detection of a margin line on the satellite brackets, using only one camera. Reference [16] used a gradient-based edge line detection method for finding the satellites brackets and acquiring its relative position and attitude.

### 1.2. Computer Vision Algorithms

An important computer vision technique needed for relative pose estimation is feature detection and matching. This method searches images for visual information, which can be utilized to identify objects in other images, while being partially or fully invariant to scaling, translation, orientation, affine distortion, illumination changes, clutter and partial occlusion. There are numerous feature point detection, description and matching methods [17].

Lowe [18] proposed the Scale Invariant Feature Transform (SIFT), which is invariant to orientation, translation, and scale. It describes each feature point using 128-element long vectors, which are used to determine matching between features in different images. These vectors are also referred to as *descriptors*. Bay, Ess, Tuytelaars and Van Gool [19] proposed the Speeded-Up Robust Features (SURF) algorithm, which is faster and more efficient than SIFT. The task of finding corresponding feature points between two images is transformed into matching descriptors from two groups. It is done by using the nearest neighbour ratio matching strategy [17,18,19], and a technique called Random Sample Consensus (RANSAC) [17,20,21], which is used to eliminate outliers.

Another important problem in computer vision is recognition of an unknown target in a given image. An unknown target is an object with a high uncertainty concerning its shape and location in the image. Jigalin and Gurfil [10] presented a method of segmentation and identification of an unknown target out of a group of potential targets in an image. They assumed some rough characteristics of the target’s shape and location, and that the target can be distinguished from the background. A series of morphological image processing operators were applied on the image in order to distinguish all the potential targets from their background. Then, the morphological properties of the target candidates were compared using a cost function, and a “best” target candidate was selected.

In the current research, the target recognition algorithm developed in Ref. [10] is expanded by checking if the “best” target meets certain criteria in order to be considered as a legitimate target. With the modified algorithm, the assumption that the target is always in the image is redundant. Moreover, this research is aimed at developing and implementing the mentioned computer vision algorithms using a specialized testbed. For a scalability analysis of the effects of larger distances on the computer vision algorithms, the reader is referred to Ref. [22].

### 1.3. Relative State Estimators

After the target was detected, it is desired to estimate its relative position and velocity with respect to the chaser. To this end, an estimator is required. In this research, three estimators, EKF, UKF and Converted Measurement Kalman Filter (CMKF), are compared and examined using experimental data.

The CMKF is less known compared to the EKF and UKF. The main idea in CMKF is to convert the measurement equation into a linear equation by an algebraic manipulation. The noise models in the converted equations are not necessarily identical to the noise model in the original equations, and, as a result, a biased estimation might occur. The CMKF was first developed by Lerro and Bar-Shalom [23] for tracking systems that measure the position of targets in polar coordinates. They also added bias compensation components, referred to as “Debiased CMKF”. Later on, Suchomski [24] proposed a three-dimensional version of this filter. A second version of the CMKF was proposed by Xiaoquan, Yiyu and Bar-Shalom [25], which was referred to as “Unbiased CMKF”. It included different bias compensation components. Later on, it was also developed using a different bias compensation technique [26].

An important issue with implications on the estimation process is the Depth Error Equation (DEE), which is an analytical equation that yields an approximation of the Line-of-Sight (LOS) vector’s depth component. The DEE can be expanded to all of the LOS components, and is referred to as Relative Position Error Equations (RPEE). The RPEE yields an analytical approximation of the expected errors of the relative position components. Gallup, Frahm, Mordohai and Pollefeys [27] investigated the DEE with changing baseline and resolution in order to compute a depth map over a certain volume with a constant depth error. Oh et al. [28] evaluated the DEE via experiments.

A direct solution of the stereo measurement model is referred to as Converted Measurements (CMS). The CMS are used for initialization of the estimators, and as a reliability check for each measurement. That is, if the CMS and the state estimation are too dissimilar, than the measurement’s validity should be questioned. In the case of stereovision with two horizontally positioned and vertically aligned cameras, the measurement model is comprised of four algebraic, coupled, overdetermined and non-linear equations. By rearranging these equation, the model’s non-linearity can be eliminated at the cost of certain numerical issues. In this case, the CMS can be attained by various methods, such Least Squares (LS) [6] and Total Least Squares (TSL) [29].

### 1.4. Research Contributions

The current research is an experimental effort, in which non-cooperative target tracking methods using stereovision measurements are developed. Real-time closed-loop line-of-sight control based on stereovision measurements only is developed and evaluated. In particular, our main contribution is the development of a stereovision tracking algorithm of non-cooperative targets suitable for real-time implementation. Moreover, we examine different estimation schemes, and evaluate their real-time performance. An emphasis is given on devising efficient methods for initializing the relative position estimators based on stereovision measurements.

The remainder of this paper is organized as follows. Section 2 introduces the computer vision models and algorithms used throughout this study. In Section 3, the estimation models are introduced, including the CMKF, which is a novel estimator. Section 4 describes the control algorithm, which is implemented in the experiments. In Section 5 a numerical analysis of the problem described in Section 3.4 is conducted. Section 6 describes the Distributed Space Systems Laboratory (DSSL) hardware, the experimental system, and three experiments, which implement the algorithms developed in this research. Section 7 contains the conclusions of this work.

Notice that Section 2, Section 3 and Section 5 address a 3D problem, while Section 4 addresses a 2D problem. The reason for that is that the estimation algorithms are designed with the intention to be general, while the control algorithm has to be adjusted to the 2D setup of the robots in the DSSL. As a result, the control algorithm uses only the relevant 2D components of the estimated 3D quantities.

## 2. Computer Vision Models and Algorithms

### 2.1. Pinhole Model

Consider *N* cameras, which are only separated by translation, and have no relative rotation, positioned at *N* different fixed points in a three-dimensional space, facing the same direction, and sharing a common image plane. A reference coordinate system is denoted by $\left\{x_{0},y_{0},z_{0}\right\}$. The camera-fixed coordinates are denoted by $\left\{x_{i},y_{i},z_{i}\right\}$. The reference coordinate system and the camera-fixed coordinate systems are rotating together and are facing the same direction at all times, so no transformation is needed between them. The vectors $\vec{b_{i}}\;\left(i=1,...,N\right)$ connect the reference coordinate system with the *N* camera-fixed coordinates.

**Figure 1:**
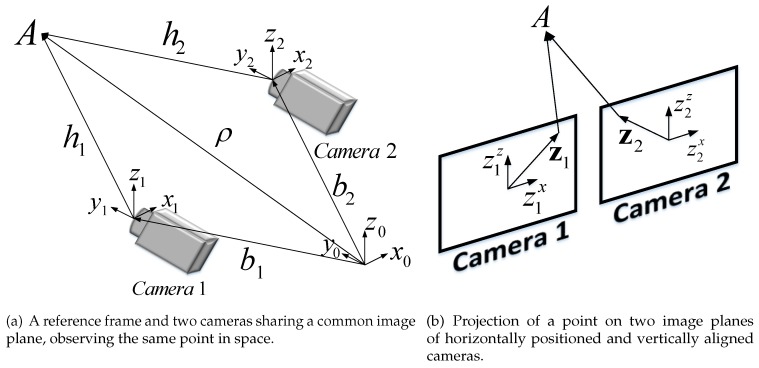
The geometry of stereovision.

The vector $\vec{\rho }$ connects $\left\{x_{0},y_{0},z_{0}\right\}$ with an arbitrary point in space, *A*. Assuming *A* appears in the Fields of View (FOV) of all the cameras, the vectors $\vec{h_{i}}\;\left(i=1,...,N\right)$ connect each camera’s respective coordinate origin with point *A*. Without loss of generality, it is assumed that all the cameras are facing the *y* direction of their respective coordinates. This geometry is depicted in Figure 1a.

Each camera’s image plane has a planar coordinate system $\left\{z_{i}^{x},z_{i}^{z}\right\}$, whose origin is positioned in the center of each image plane, as depicted in Figure 1b. The vectors ${\vec{z}}_{i}$ connect the center of the image plane of camera *i* to the projection of point *A* on the image plane. They have a horizontal component $z_{i}^{x}$, a vertical component $z_{i}^{z}$, and are measured in pixels.

It is important to distinguish between the coordinate frames $\left\{x_{i},y_{i},z_{i}\right\}$ and $\left\{z_{i}^{x},z_{i}^{z}\right\}$. The former is a three-dimensional coordinate frame, located in a three-dimensional space, in which distances are dimensional; the latter is a two-dimensional coordinate frame, located on the image plane, in which distances are measured in pixels. The pinhole camera model [17] yields the following mathematical relation for each camera:

(1) $$\left(\begin{array}{c} z_{i}^{x}\\ z_{i}^{z} \end{array}\right)=\frac{1}{h_{i}^{y}}\left(\begin{array}{c} f_{i}^{x}h_{i}^{x}\\ f_{i}^{z}h_{i}^{z} \end{array}\right)$$

where $f_{i}^{x}$ and $f_{i}^{z}$ are the focal lengths of each camera (measured in pixels) in the *x* and *z* directions, respectively, and $h_{i}^{x},h_{i}^{y},h_{i}^{z}$ are the components of vector ${\vec{h}}_{i}$ in the reference coordinate system. From Figure 1a, it can be seen that

(2) $$\vec{\rho }={\vec{b}}_{i}+{\vec{h}}_{i}$$

Therefore, the *non-linear projection equations* are

(3) $$\left(\begin{array}{c} z_{i}^{x}\\ z_{i}^{z} \end{array}\right)=\frac{1}{\rho_{y}-b_{i}^{y}}\left(\begin{array}{c} f_{i}^{x}\left(\rho_{x}-b_{i}^{x}\right)\\ f_{i}^{z}\left(\rho_{z}-b_{i}^{z}\right) \end{array}\right)$$

These equations can also be rearranged in the following manner, which is referred to as the *linear projection equations*:

(4) $$\left(\begin{array}{ccc} 1 & -z_{i}^{x}/f_{i}^{x} & 0\\ 0 & -z_{i}^{z}/f_{i}^{z} & 1 \end{array}\right)\left(\begin{array}{c} \rho_{x}\\ \rho_{y}\\ \rho_{z} \end{array}\right)=\left(\begin{array}{ccc} 1 & -z_{i}^{x}/f_{i}^{x} & 0\\ 0 & -z_{i}^{z}/f_{i}^{z} & 1 \end{array}\right)\left(\begin{array}{c} b_{i}^{x}\\ b_{i}^{y}\\ b_{i}^{z} \end{array}\right)$$

When all the cameras share the same plane, meaning $b_{y}^{i}=0$, Equations (3) and (4) yield

(5) $$\left(\begin{array}{c} z_{i}^{x}\\ z_{i}^{z} \end{array}\right)=\frac{1}{\rho_{y}}\left(\begin{array}{c} f_{i}^{x}\left(\rho_{x}-b_{i}^{x}\right)\\ f_{i}^{z}\left(\rho_{z}-b_{i}^{z}\right) \end{array}\right)\;i=1,...,N$$

(6) $$\left(\begin{array}{ccc} 1 & -z_{i}^{x}/f_{i}^{x} & 0\\ 0 & -z_{i}^{z}/f_{i}^{z} & 1 \end{array}\right)\left(\begin{array}{c} \rho_{x}\\ \rho_{y}\\ \rho_{z} \end{array}\right)=\left(\begin{array}{c} b_{i}^{x}\\ b_{i}^{z} \end{array}\right)\;i=1,...,N$$

### 2.2. Image Resizing

Consider an image with the dimensions $X_{1}$ and $Y_{1}$ and focal lengths $f_{base}^{x}$, $f_{base}^{z}$ in the *x* and *y* directions, respectively. Assume that it is desired to resize that image to the dimensions $X_{2}$ and $Y_{2}$. To that end, $R_{F}$ is defined as the *Resize Factor*. For simplicity, we assume that the image is resized while keeping the aspect ratio constant. The relation between $X_{1}$, $X_{2}$, $Y_{1}$, $Y_{2}$ and $R_{F}$ is

(7) $$X_{2}=R_{F}\;X_{1}\;,\;Y_{2}=R_{F}\;Y_{1}$$

When images are resized, their focal length should be corrected correspondingly,

(8) $$\begin{array}{c} f^{x}=R_{F}f_{base}^{x}\\ f^{z}=R_{F}f_{base}^{z} \end{array}$$

where $f^{x}$ and $f^{y}$ are the focal lengths of the resized image.

### 2.3. Relative Position Measurement Error Approximation

The error of the measured relative position ${\vec{\rho }}_{m}$ for a simple case of two horizontally positioned and vertically aligned cameras, is approximated in the following manner. It is assumed that the focal lengths of the cameras are equal,

(9) $$f_{1}^{x}=f_{1}^{z}=f_{2}^{x}=f_{2}^{z}=f$$

**Figure 2:**
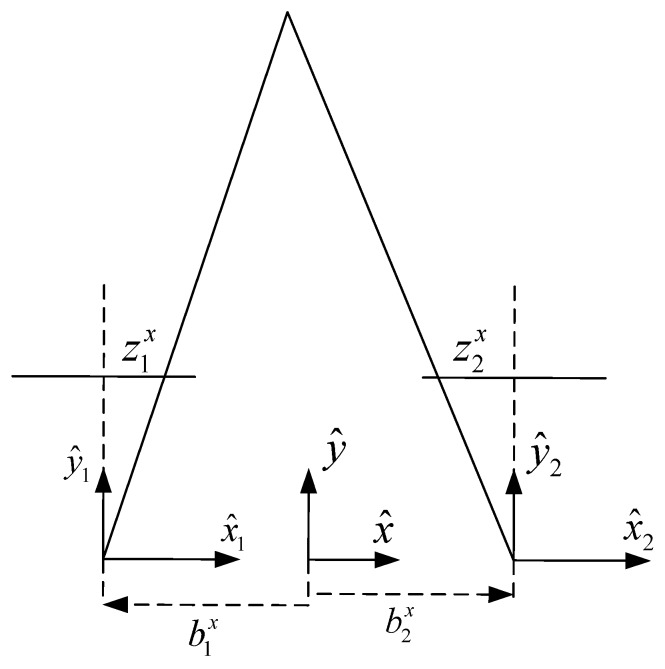
A two dimensional geometry of two aligned cameras and a point’s projection on their respective image planes.

In Figure 2, depicting the geometry of aligned cameras, it can be seen that $z_{1}^{x}>0$, $z_{2}^{x}<0$, $b_{1}^{x}<0$, $b_{2}^{x}>0$. Therefore,

(10) $$\begin{array}{c} b=b_{2}^{x}-b_{1}^{x}>0\\ d=z_{1}^{x}-z_{2}^{x}>0 \end{array}$$

where *b* is the distance between the cameras, also referred to as *baseline*, and *d* is the *disparity* [17,21]. The linear projection Equations (6) for this case become

(11) $$\left[\begin{array}{ccc} 1 & -\frac{1}{f}z_{1}^{x} & 0 \end{array}\right]{\left[\begin{array}{ccc} \rho_{x} & \rho_{y} & \rho_{z} \end{array}\right]}^{T}=b_{1}^{x}$$

(12) $$\left[\begin{array}{ccc} 1 & -\frac{1}{f}z_{2}^{x} & 0 \end{array}\right]{\left[\begin{array}{ccc} \rho_{x} & \rho_{y} & \rho_{z} \end{array}\right]}^{T}=b_{2}^{x}$$

(13) $$\left[\begin{array}{ccc} 0 & -\frac{1}{f}z_{1}^{z} & 1 \end{array}\right]{\left[\begin{array}{ccc} \rho_{x} & \rho_{y} & \rho_{z} \end{array}\right]}^{T}=0$$

(14) $$\left[\begin{array}{ccc} 0 & -\frac{1}{f}z_{2}^{z} & 1 \end{array}\right]{\left[\begin{array}{ccc} \rho_{x} & \rho_{y} & \rho_{z} \end{array}\right]}^{T}=0$$

Equations (11) and (12) are referred to as the *Horizontal Equations*, while Equations (13) and (14) are referred to as the *Vertical Equations*. These equations can be solved using the Combined Vertical Equations (CVE) method. The CVE approach, developed in this research, addresses the specific problem of stereovision with two horizontally positioned and vertically aligned cameras. According to this approach, it is noticed that the vertical equations in both cameras are identical up to measurement errors. Therefore, to render the linear system determined, the vertical equations are averaged into one equation. Consequently, a solution can be achieved by simply inverting the matrix in the left-hand side of Horizontal Equations, which becomes a 3×3 matrix. The solutions according to the CVE method for $\rho_{x}$, $\rho_{y}$, $\rho_{z}$ are given by

(15) $$\begin{array}{c} \rho_{x}=\frac{z_{1}^{x}b_{2}^{x}-z_{2}^{x}b_{1}^{x}}{z_{1}^{x}-z_{2}^{x}}\\ \rho_{y}=\frac{f(b_{2}^{x}-b_{1}^{x})}{\left(z_{1}^{x}-z_{2}^{x}\right)}\\ \rho_{z}=\frac{\left(z_{1}^{z}+z_{2}^{z}\right)\left(b_{2}^{x}-b_{1}^{x}\right)}{2(z_{1}^{x}-z_{2}^{x})}=\frac{z_{1}^{z}+z_{2}^{z}}{2f}\rho_{y} \end{array}$$

The horizontal error, depth error and vertical error, denoted by $\Delta \rho_{x}$, $\Delta \rho_{y}$, $\Delta \rho_{z}$, respectively are defined as

(16) $$\begin{array}{c} \Delta \rho_{i}=\sqrt{{\left(\frac{\partial \rho_{i}}{\partial z_{1}^{x}}\Delta z_{1}^{x}\right)}^{2}+{\left(\frac{\partial \rho_{i}}{\partial z_{2}^{x}}\Delta z_{2}^{x}\right)}^{2}+{\left(\frac{\partial \rho_{i}}{\partial z_{1}^{z}}\Delta z_{1}^{z}\right)}^{2}+{\left(\frac{\partial \rho_{i}}{\partial z_{2}^{z}}\Delta z_{2}^{z}\right)}^{2}}\\ i=\{x,y,z\} \end{array}$$

where $\Delta z_{1}^{x}$, $\Delta z_{2}^{x}$, $\Delta z_{1}^{z}$, $\Delta z_{2}^{z}$ are the errors in $z_{1}^{x}$, $z_{2}^{x}$, $z_{1}^{z}$, $z_{2}^{z}$, respectively. Furthermore, it is assumed that

(17) $$\begin{array}{c} \Delta z\approx \Delta z_{1}^{x}\approx \Delta z_{2}^{x}\approx \Delta z_{1}^{z}\approx \Delta z_{2}^{z} \end{array}$$

By combining Equations (15)–(17), the following expressions are obtained:

(18) $$\begin{array}{c} \Delta \rho_{x}=\frac{\sqrt{{\left(z_{1}^{x}\right)}^{2}+{\left(z_{2}^{x}\right)}^{2}}\left(b_{2}^{x}-b_{1}^{x}\right)}{{\left(z_{1}^{x}-z_{2}^{x}\right)}^{2}}\Delta z=\frac{\rho_{y}}{bf}\sqrt{{\left(\rho_{x}-b_{1}^{x}\right)}^{2}+{\left(\rho_{x}-b_{2}^{x}\right)}^{2}}\Delta z\\ \Delta \rho_{y}=\frac{\sqrt{2}f\left(b_{1}^{x}-b_{2}^{x}\right)}{{\left(z_{1}^{x}-z_{2}^{x}\right)}^{2}}\Delta z=\frac{\sqrt{2}fb}{d^{2}}\Delta z=\frac{\rho_{y}^{2}}{bf}\sqrt{2}\Delta z\\ \Delta \rho_{z}=\sqrt{\frac{{\left(b_{2}^{x}-b_{1}^{x}\right)}^{2}}{2{\left(z_{1}^{x}-z_{2}^{x}\right)}^{2}}+\frac{{\left(z_{1}^{z}+z_{2}^{z}\right)}^{2}{\left(b_{2}^{x}-b_{1}^{x}\right)}^{2}}{2{\left(z_{1}^{x}-z_{2}^{x}\right)}^{4}}}\Delta z=\sqrt{\frac{\rho_{y}^{2}}{2f^{2}}+\frac{2\rho_{y}^{2}\rho_{z}^{2}}{b^{2}f^{2}}}\Delta z=\frac{\rho_{y}}{bf}{\left(\frac{b^{2}}{2}+2\rho_{z}^{2}\right)}^{\frac{1}{2}}\Delta z \end{array}$$

Finally, the relative position vector is approximated as

(19) $$\vec{\rho }=\left[\begin{array}{c} \rho_{x}\\ \rho_{y}\\ \rho_{z} \end{array}\right]\approx \left[\begin{array}{c} \rho_{m}^{x}\\ \rho_{m}^{y}\\ \rho_{m}^{z} \end{array}\right]+\left[\begin{array}{c} \Delta \rho_{x}\\ \Delta \rho_{y}\\ \Delta \rho_{z} \end{array}\right]={\vec{\rho }}_{m}+\Delta \vec{\rho }$$

where ${\vec{\rho }}_{m}$ is the *converted measurement*, calculated using Equations (11)–(14), and $\Delta \vec{\rho }$ is the additive error vector.

### 2.4. Computer Vision Algorithms

To utilize the stereovision camera as a sensor, the target has to be identified in the scene, following the extraction of its position in the image plane in all cameras. To that end, a methodology which relies on feature detection and matching, image segmentation and target recognition algorithms is used.

#### 2.4.1. Feature Detection and Matching

Feature detection and matching [17] is used for matching the target in all cameras, by detecting feature points in the images and assigning to each point a descriptor with encoded visual information. Consequently, it is possible to find the same point in different images by matching the descriptors numerically.

Over the years, different kinds of feature point detection methods were developed. The most well known is SIFT [18], and there are others such as SURF [19], Maximally Stable Extremal Regions (MSER) [30], Features from Accelerated Segment Test (FAST) [31] and Oriented fast and Rotated Brief (ORB) [32]. Each method relies on a different geometric concept, and has different properties, such as computational effort and reliability.

In this research, SURF was used. The computational effort of SURF is significantly smaller than SIFT, which was used by Jigalin and Gurfil [10]. The feature matching methods used herein are:

1. Nearest Neighbour—The descriptors of the feature points of all the cameras are matched by comparing them using Euclidian distances. The reliability of the matches increases by eliminating ambiguous matches, which are defined as matches that have a low ratio between their distance and the distance of the second-closest neighbor (see [18]).
2. Fundamental Matrix Estimation Using RANSAC [21]—This method eliminates the outliers of the Nearest Neighbour method by applying geometric constraints.
3. Slope Cut Off—This method eliminates more outliers by enforcing geometric consistency. It places the stereovision images side by side and stretches lines between all the matched points. For every object, all the lines should have a similar slope, so the lines should not cut each other. The matches of lines that do not adhere to these rules are declared as outliers and rejected.

#### 2.4.2. Image Segmentation

The following image segmentation method [10] is used for locating all the target candidates in the image plane. A target candidate’s projection on the image plane is assumed to be a notable blob. With this assumption, the images from all the cameras are subjected to the following procedures

1. The images are processed by a Sobel edge detector [17], which returns binary images of the edges in the scene.
2. The binary images are filtered in order to clean noisy pixels.
3. The images are processed through an image dilation operator, which expands the outlines of the objects in the scene. This operation closes all the gaps in the objects’ outlines.
4. After the gaps in the objects’ outlines are closed, the objects, which are closed outlined blobs, are filled.
5. Finally, the images are processed by an erosion operator, which diminishes all the blobs in the images. This operation leaves only the most significant blobs.

All the remaining separate blobs are considered as target candidates.

#### 2.4.3. Target Recognition

The next step is to determine which of the obtained target candidates is the real one. In order to do so, the following method is used. The method starts by calculating the area, solidity and distance of each target candidate’s region. *Area* is the number of pixels in each region. *Solidity* is the area fraction of the region with respect to its convex hull (0<Solidity<1). *Distance* is the mean distance of SURF feature points in each region.

The distance value for each feature point is simply the *y* component of the LOS vector $\vec{\rho }$ to each feature point. These vectors are obtained by solving Equation (6) for each feature point. An important issue that has to be dealt with, is the method which is used to solve Equation (6). Among other methods, it can be solved using LS or TLS, which were mentioned above. A comparison is given in Section 5.

The properties (*Area*, *Solidity* and *Distance*) of each target candidate has to be bounded within certain upper and lower bounds. Each target candidate, whose properties are not limited to these bounds, is neglected. Among the target candidates, which meet the previous criteria, the true target is selected using the relationship

(20) $$Target\;Region=\max_{i=1}^{N_{c}}\left(\frac{\sqrt{Area_{i}}\cdot Solidity_{i}}{Distance_{i}}\right)$$

where $N_{c}$ is the number of the target candidates. It can be seen that this formula gives a preference to larger, closer and more convex objects using a non-dimensional value.

## 3. Estimation of the Relative State

### 3.1. Process Model

We define I as a cartesian right-hand inertial reference frame. The state vector, $\vec{x}$, contains the relative position and velocity between the chaser and the target in the inertial coordinate frame I,

(21) $$\vec{x}={\left[\begin{array}{cc} {\vec{\rho }}^{T} & {\vec{\dot{\rho }}}^{T} \end{array}\right]}^{T}$$

To write a process model, it is assumed that the target’s mass, inertia, applied forces and torques are unknown; therefore, an exact dynamical model, which describes the relative state between the chaser and the target, is quite complicated to derive. Instead, a white-noise acceleration model [6] can be implemented. This model is less accurate, but also does not require much information regarding the target. The continuous process model is written as

(22) $$\dot{\vec{x}}=A\vec{x}+\vec{\nu }\;,\;A=\left[\begin{array}{cc} 0_{3\times 3} & I_{3\times 3}\\ 0_{3\times 3} & 0_{3\times 3} \end{array}\right]$$

where $\vec{\nu }\left(t\right)\in R^{6\times 1}$ is a white Gaussian process noise vector defined as

(23) $$\vec{\nu }={\left[\begin{array}{cccccc} 0 & 0 & 0 & \nu_{\dot{x}} & \nu_{\dot{y}} & \nu_{\dot{z}} \end{array}\right]}^{T}$$

satisfying

(24) $$\begin{array}{c} E\left[\vec{\nu }\left(t\right)\right]=0\;,\;E\left[\vec{\nu }\left(t\right){\vec{\nu }}^{T}\left(t+\tau \right)\right]=\vec{q_{\nu }}\left(t\right)\delta \left(t-\tau \right)=Q_{c} \end{array}$$

with $Q_{c}$ being the power spectral density, and

(25) $$\vec{q_{\nu }}={\left[\begin{array}{cccccc} 0 & 0 & 0 & \sigma_{x}^{2} & \sigma_{y}^{2} & \sigma_{z}^{2} \end{array}\right]}^{T}$$

The next step is to write the discrete-time process model with a sampling period Δt,

(26) $${\vec{x}}_{k+1}=\Phi {\vec{x}}_{k}+{\vec{\nu }}_{k}$$

where Φ is the discrete state transition matrix,

(27) $$\Phi =e^{A\Delta t}=\left[\begin{array}{cc} I_{3} & I_{3}\Delta t\\ 0_{3\times 3} & I_{3} \end{array}\right]$$

The discrete process noise vector ${\vec{\nu }}_{k}$ satisfies

(28a) $$\begin{array}{c} E({\vec{\nu }}_{k})=0 \end{array}$$

(28b) $$\begin{array}{c} \begin{array}{c} E\left({\vec{\nu }}_{k}{\vec{\nu }}_{k}^{T}\right)=Q=\int_{0}^{\Delta t}e^{A\tau }Q_{c}e^{A^{T}\tau }d\tau \\ Q=\left[\begin{array}{cccccc} \frac{\sigma_{x}^{2}{\Delta t}^{3}}{3} & 0 & 0 & \frac{\sigma_{x}^{2}{\Delta t}^{2}}{2} & 0 & 0\\ 0 & \frac{\sigma_{y}^{2}{\Delta t}^{3}}{3} & 0 & 0 & \frac{\sigma_{y}^{2}{\Delta t}^{2}}{2} & 0\\ 0 & 0 & \frac{\sigma_{z}^{2}{\Delta t}^{3}}{3} & 0 & 0 & \frac{\sigma_{z}^{2}{\Delta t}^{2}}{2}\\ \frac{\sigma_{x}^{2}{\Delta t}^{2}}{2} & 0 & 0 & \sigma_{x}^{2}\Delta t & 0 & 0\\ 0 & \frac{\sigma_{y}^{2}{\Delta t}^{2}}{2} & 0 & 0 & \sigma_{y}^{2}\Delta t & 0\\ 0 & 0 & \frac{\sigma_{z}^{2}{\Delta t}^{2}}{2} & 0 & 0 & \sigma_{z}^{2}\Delta t \end{array}\right] \end{array} \end{array}$$

where *Q* is the covariance matrix.

### 3.2. Measurement Model

The non-linear projection equations (Equation (5)) for *N* cameras satisfy the relation

(29) $$\vec{z}=\vec{h}\left(\vec{x}\right)+\vec{w}$$

where

(30) $$\begin{array}{c} \vec{z}={\left[\begin{array}{ccccc} z_{1}^{x} & z_{1}^{z} & \ldots & z_{N}^{x} & z_{N}^{z} \end{array}\right]}^{T} \end{array}$$

(31) $$\begin{array}{c} \vec{h}\left(\vec{x}\right)=\frac{1}{\rho_{y}}\left[\begin{array}{c} f_{1}^{x}\left(\rho_{x}-b_{1}^{x}\right)\\ f_{1}^{z}\left(\rho_{z}-b_{1}^{z}\right)\\ \vdots \\ f_{N}^{x}\left(\rho_{x}-b_{N}^{x}\right)\\ f_{N}^{z}\left(\rho_{z}-b_{N}^{z}\right) \end{array}\right] \end{array}$$

$\vec{z}$ denotes the coordinates in pixels of the Center of Projection (COP) of the target relative to the center of the images. $f_{1}^{x},f_{1}^{z},...,f_{N}^{x},f_{N}^{z}$ are the focal lengths in pixels for each camera and each direction separately. $\vec{w}$ is a zero-mean Gaussian measurement noise vector and *R* is its covariance matrix,

(32) $$E\left({\vec{w}}_{k}\right)=0\;,\;E\left({\vec{w}}_{k}{\vec{w}}_{k}^{T}\right)=R$$

### 3.3. CMKF

The CMKF [23,24,25,26] is a less common filter than the EKF and the UKF, and it cannot be implemented on all non-linear systems; only the measurement equations are allowed to be non-linear (the process equations are compelled to be linear) and not all non-linearities can be dealt with. The main idea in the CMKF is to rearrange the non-linear measurement equations into linear equations.

The CMKF is a linear filter, and therefore has a stability proof, which leads to robustness to different initial conditions and to large time increments. On the other hand, the noise in the rearranged measurement equations is not necessarily white, and it may be difficult to determine its statistics. An inaccurate assessment of the noise statistics can result in a biased estimation of the state.

The CMKF fits quite well to the case of relative position estimation using stereovision with no a-priori information. The process equation is linear (Equation (26)) and the non-linearity in the measurement equation (Equation (5)) can be dealt with (Equation (6)).

Equation (6) shows that

(33) $$\left(\begin{array}{ccc} 1 & -z_{1}^{x}/f_{1} & 0\\ 0 & -z_{1}^{z}/f_{1} & 1\\ \vdots & & \\ 1 & -z_{N}^{x}/f_{N} & 0\\ 0 & -z_{N}^{z}/f_{N} & 1 \end{array}\right){\vec{\rho }}_{m}=\left(\begin{array}{c} b_{x}^{1}\\ b_{z}^{1}\\ \vdots \\ b_{x}^{N}\\ b_{z}^{N} \end{array}\right)\;,\;{\vec{\rho }}_{m}={\left(\begin{array}{c} \rho_{x}\\ \rho_{y}\\ \rho_{z} \end{array}\right)}_{m}$$

This is a linear system, $A{\vec{\rho }}_{m}=\vec{b}$. The unknown vector ${\vec{\rho }}_{m}$ can be evaluated by solving Equation (33) using different methods, as discussed in Section 5.

Although the vector ${\vec{\rho }}_{m}$ does not necessarily contain additive noise, it was approximated in Section 2.3 using a linear model

(34) $${\vec{\rho }}_{m}=f\left(\vec{\rho },\vec{w}\right)\approx \vec{\rho }+\vec{w}=\left[\begin{array}{cc} I_{3\times 3} & 0_{3\times 3} \end{array}\right]\vec{x}+\vec{w}$$

Equation (34) is the CMKF measurement equation, where $\vec{w}$ is a zero-mean measurement noise vector and *R* is its covariance matrix.

(35) $$E\left({\vec{w}}_{k}\right)=0\;,\;E\left({\vec{w}}_{k}{\vec{w}}_{k}^{T}\right)=R=\left[\begin{array}{ccc} \sigma_{\rho_{x}}^{2} & 0 & 0\\ 0 & \sigma_{\rho_{y}}^{2} & 0\\ 0 & 0 & \sigma_{\rho_{z}}^{2} \end{array}\right]$$

where $\sigma_{\rho_{x}}$, $\sigma_{\rho_{y}}$, $\sigma_{\rho_{z}}$ are approximated using Equation (18),

(36) $$\begin{array}{c} \sigma_{\rho_{x}}=\frac{\rho_{y}}{bf}\sqrt{{\left(\rho_{x}-b_{1}^{x}\right)}^{2}+{\left(\rho_{x}-b_{2}^{x}\right)}^{2}}\;\Delta z\;,\;\sigma_{\rho_{y}}=\frac{\rho_{y}^{2}}{bf}\sqrt{2}\;\Delta z\;,\;\sigma_{\rho_{z}}=\frac{\rho_{y}}{bf}{\left(\frac{b^{2}}{2}+2\rho_{z}^{2}\right)}^{\frac{1}{2}}\Delta z \end{array}$$

Δz is the standard deviation of the coordinates of the centroid of the projection of the target onto the image plane, assuming that the standard deviation Δz is the same in the *x* and *z* directions in both cameras. The rest of the CMKF equations are standard Kalman Filter (KF) equations.

The full details of the CMKF algorithm are described in Algorithm 1, where *P*, *Q*, *R* are the state covariance, process noise covariance and measurement noise covariance respectively, Φ is the discrete state-transition matrix, *K* is the gain matrix, *k* is the time step index and ${\vec{\rho }}_{m}$ is an estimation of the LOS vector $\vec{\rho }$.

**Algorithm 1** CMKF1:**Initialization**:2:
3:
4:  ${\vec{\hat{x}}}_{0}=E\left[{\vec{x}}_{0}\right]$5:
6:
7:  $P_{0}=E\left[\left({\vec{x}}_{0}-{\vec{\hat{x}}}_{0}\right){\left({\vec{x}}_{0}-{\vec{\hat{x}}}_{0}\right)}^{T}\right]$8:
9:
10:**while** Target is within FOV **do**11:
12:
13:  **Time Propagation**:14:
15:
16:    ${\vec{\hat{x}}}_{k+1|k}=\Phi {\vec{\hat{x}}}_{k|k}$17:
18:
19:    $P_{k+1|k}=\Phi P_{k|k}\Phi^{T}+Q$20:
21:
22:  **Create Pseudo-Measurements**:23:
24:
25:    ${\vec{z}}_{k}={\left(\vec{\rho_{m}}\right)}_{k}$27:
28:    $H=\left[\begin{array}{cc} I_{3\times 3} & 0_{3\times 3} \end{array}\right]$29:
30:
31:  **Measurement Update**:32:
33:
34:    $K_{k}={\left(P_{k+1|k}H^{T}\right)}^{-1}\left(HPH^{T}+R\right)$35:
36:
37:    ${\vec{\hat{x}}}_{k+1|k+1}={\vec{\hat{x}}}_{k+1|k}+K_{k}\cdot \left({\vec{z}}_{k}-H\cdot {\vec{\hat{x}}}_{k+1|k}\right)$38:
39:
40:    $P_{k+1|k+1}=\left(I-K_{k}H\right)P_{k+1|k}{\left(I-K_{k}H\right)}^{T}+K_{k}RK_{k}^{T}$41:
42:
43:**end**
**while**

### 3.4. Filters Initialization

By using the methods described in Section 2.4, the obtained measurements are processed in order to initialize the filters through Equation (6), which can be solved using several methods, e.g., LS, TLS and CVE. These methods solve the overdetermined system of linear equations $A\vec{x}=\vec{b}$, where *A* is a matrix with more rows than columns. In the LS approach [6], there is an underlying assumption that all the errors are confined to the observation vector $\vec{b}$; its solution is $\vec{x}={\left(A^{T}A\right)}^{-1}A^{T}\vec{b}$. In the TLS approach [29] it is assumed that there are errors both in $\vec{b}$ and *A*. The CVE approach was presented in Section 2.3. In order to determine the preferred method of solution, the performance of these methods will be examined in Section 5.

### 3.5. Estimation Scheme

**Figure 3:**
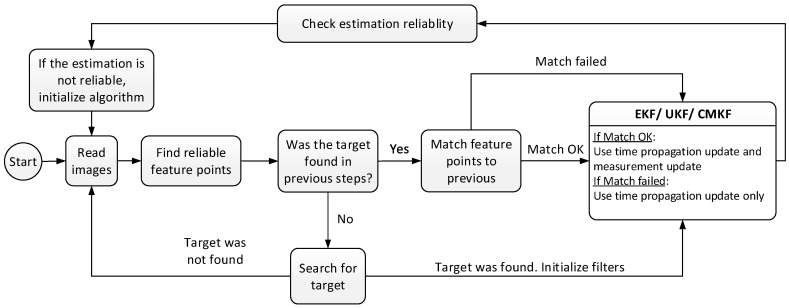
The estimation logic. This scheme consists of a filter re-initialization step, which is activated in case the estimation is no longer reliable.

The estimation scheme in Figure 3 describes the implemented algorithm. Every step, a measurement is acquired. If a target has not been found yet, or the algorithm was initialized in the last step, then a target recognition algorithm is activated. Only if a target was found by the target recognition algorithm, the different filters are initialized. After each filtering step, the algorithm checks if the estimation is reliable. Estimation is declared as non-reliable if the produced state is not reasonable in relation to the laboratory dimensions or to the state of the previous step. A non-reliable state can also be produced in case there has been too few matched feature points for several sequential steps. This happens mostly if the target has left the scene. If the state is unreliable, the algorithm is re-initialized, meaning that the filters are initialized and the target has to be found again.

## 4. Target Tracking

The LOS between the chaser and the target is the vector $\vec{\rho }$, defined in previous sections. It determines whether the target is visible to the chaser.

The direction of the LOS can be expressed using azimuth and elevation angles. In general, the tracking algorithm has to keep these two angles bounded. In the two dimensional case, only the azimuth angle has to be controlled. The bounds of these angles are determined by the camera rig’s FOV, which is determined by the cameras’ properties and the geometric arrangement.

### 4.1. FOV Azimuth Angle Bounds

**Figure 4:**
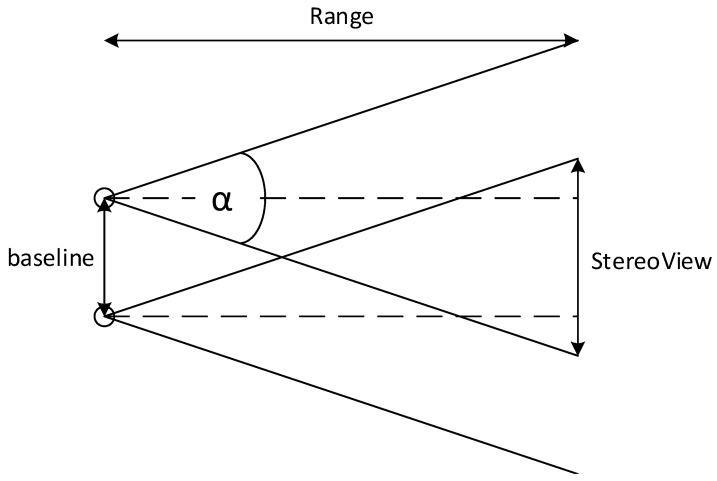
A two dimensional geometry of two aligned cameras with their combined FOV.

Figure 4 depicts two vertically aligned and horizontally positioned cameras, and their combined FOV, denoted by StereoView. α is the horizontal Angle-of-View (AOV), baseline is the distance between the cameras and Range is the distance between the cameras and the target. The following relation can be inferred:

(37) $$StereoView=2\tan\left(\frac{\alpha }{2}\right)Range-baseline$$

It can be seen that the use of stereovision diminishes the combined FOV. More specifically, enlargement of the baseline leads to a smaller StereoView. On the other hand, increasing the baseline also leads to a higher accuracy (Equation (18)). By substituting StereoView=0 into Equation (37), The minimal Range is calculated.

(38) $$Range_{min}=\frac{baseline}{2\tan\left(\frac{\alpha }{2}\right)}$$

The horizontal AOV of each camera is expressed as

(39) $$\alpha =2\arctan\left(\frac{SensorWidth}{2f}\right)=2\arctan\left(\frac{SensorWidth}{2R_{F}f_{base}}\right)$$

where *f* is the focal length. $f_{base}$ and $R_{F}$ are the basic focal length and resize factor, respectively, as defined in Section 2.2. SensorWidth is the width of the optical sensor.

**Figure 5:**
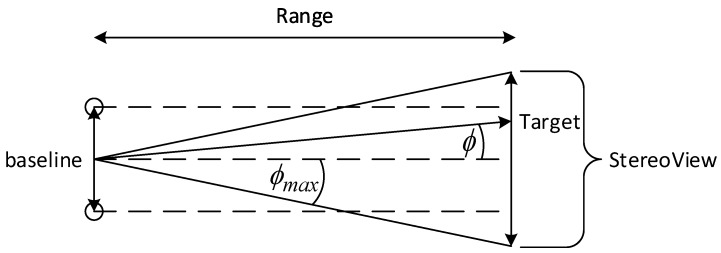
A two dimensional geometry of two aligned cameras with their horizontal angle of view bound.

Figure 5 depicts StereoView as defined in Equation (37). ϕ is the azimuth angle of the LOS vector $\vec{\rho }$. In order to maintain the target within StereoView, $\phi_{max}$ is defined as the maximal allowed azimuth angle for a certain Range. From the geometry depicted in Figure 5, $\phi_{max}$ is calculated,

(40) $$\begin{array}{c} \left|\phi \right|\leq \phi_{max}\\ \phi_{max}\left(Range\right)=\arctan\left(\frac{StereoView/2}{Range}\right) \end{array}$$

### 4.2. Tracking Algorithm

**Figure 6:**
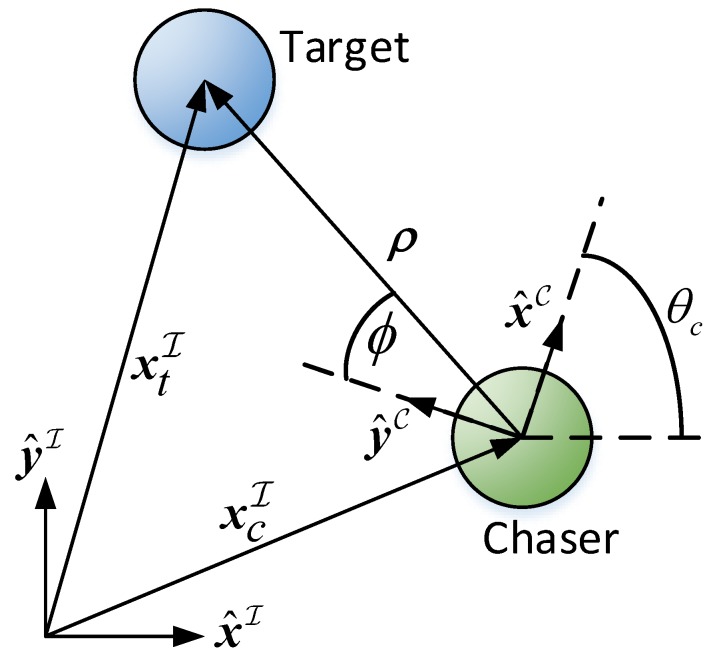
Top view of a chaser and a target, showing the azimuth angle.

Figure 6 depicts the chaser, the target and the LOS vector $\vec{\rho }$. ${\vec{x}}_{c}^{I}$ and ${\vec{x}}_{t}^{I}$ are the chaser and target’s inertial position vectors, respectively. $\theta_{c}$ is the chaser’s body angle. C is a cartesian right-hand body-fixed reference frame attached to the chaser. ${\vec{\hat{x}}}^{C}$, ${\vec{\hat{y}}}^{C}$, ${\vec{\hat{x}}}^{I}$ and ${\vec{\hat{y}}}^{I}$ are the principal axes of the chaser’s body frame and the inertial frame, respectively. Assume that the camera rig is installed on the chaser along the ${\vec{\hat{y}}}_{C}$ direction. ϕ is the azimuth angle and is calculated as

(41) $$\phi =\tan^{-1}\left[\frac{{\left({\vec{\hat{x}}}^{C}\right)}^{T}{\vec{\rho }}^{C}}{{\left({\vec{\hat{y}}}^{C}\right)}^{T}{\vec{\rho }}^{C}}\right]=\tan^{-1}\left(\frac{\rho_{x}^{C}}{\rho_{y}^{C}}\right)$$

**Figure 7:**
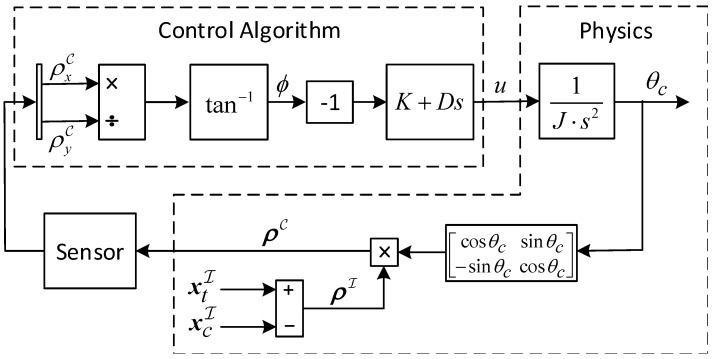
The LOS Control Algorithm.

Figure 7 depicts the LOS control algorithm. In the tracking algorithm, it is desired to maintain the target in the FOV center, or in other words, to minimize |ϕ|. It is done by a Proportional Derivative (PD) controller. The signals ${\vec{x}}_{c}^{I}$ and ${\vec{x}}_{t}^{I}$ are constantly changing and, therefore, ϕ will not converge to zero.

In Section 3.5 it was mentioned that there are cases where the state is unreliable and the estimation algorithm is initialized. In that case, there is no estimation of the vector $\vec{\rho }$, and the tracking algorithm cannot be implemented. For the sake of simplicity, in those cases, a default reference value of 0 degrees is used for $\theta_{c}$, which makes the robot look straight ahead to the direction of the target, assuming it is not moving.

### 4.3. Tracking Algorithm Stability

The closed-loop dynamics in Figure 7 can be written as

(42) $${\ddot{\theta }}_{c}+\frac{D}{J}\frac{{\left(\rho_{x}^{I}\right)}^{2}+{\left(\rho_{y}^{I}\right)}^{2}}{{\left(\rho_{y}^{I}\cos\left(\theta_{c}\right)-\rho_{x}^{I}\sin\left(\theta_{c}\right)\right)}^{2}}{\dot{\theta }}_{c}+\frac{K}{J}\frac{\rho_{x}^{I}\cos\left(\theta_{c}\right)+\rho_{y}^{I}\sin\left(\theta_{c}\right)}{\rho_{y}^{I}\cos\left(\theta_{c}\right)-\rho_{x}^{I}\sin\left(\theta_{c}\right)}=0$$

where *K* and *D* are the PD controller proportional and derivative gains, respectively. *J* is the polar moment of inertia of the chaser. Notice that the properties of the chaser are assumed to be known. The stability analysis will be carried out around an equilibrium where the chaser is looking directly at the target. For simplicity, it is also assumed that the chaser and the target share the same $x^{I}$ coordinate. In other words,

(43) $${\left(\rho_{x}^{I}\right)}_{eq}=0\;;\;{\left(\theta_{c}\right)}_{eq}=0$$

At this equilibrium, Equation (42) yields

(44) $${\ddot{\theta }}_{c}+\frac{D}{J}{\dot{\theta }}_{c}+\frac{K}{J}\theta_{c}=0$$

It can be seen that for K,D>0 (J>0 because it is a moment of inertia), Equation (44) represents an asymptotically stable system.

This system is also asymptotically stable for a more general case, where the chaser is looking directly to the target, but the LOS vector is not aligned with the inertial coordinate system I, that is,

(45) $${\left(\rho_{x}^{I}\right)}_{eq}\neq 0\;;\;{\left(\theta_{c}\right)}_{eq}\neq 0\;;\;{\left(\rho_{x}^{C}\right)}_{eq}=0\;;\;\phi_{eq}=0$$

In this case, in order to prove stability, it is required to define a new inertial coordinate frame, which is rotated by ${\left(\theta_{c}\right)}_{eq}$ with respect to I. In the rotated inertial coordinate frame, Equation (43) is satisfied and the stability proof holds.

## 5. Numerical Study

To initialize the filters (Section 3.4) and the distance value for each feature point (Section 2.4.3), it is required to solve Equation (6). In this section, a numerical evaluation will compare the solutions of Equation (6) using the LS, TLS and CVE approaches.

### 5.1. Static Problem Description

Consider a static object, which is viewed by a camera rig with either 2 or 4 cameras. In the case of 2 cameras, the cameras are vertically aligned and horizontally positioned with a baseline of length *b*. In the case of 4 cameras, the cameras are located at the vertices of a square with edges of length *b*.

Each camera acquires measurements of the viewed object according to Equation (5). Each measurement is corrupted with a zero mean Gaussian noise with a standard deviation ΔZ and is rounded to the closest integer (because the measurements are in pixels). The noisy measurements are then used for writing Equation (6), which is solved using LS and TLS. In the case of 2 cameras, CVE is also used.

This routine is carried out in 5000 Monte-Carlo runs, which produce estimations for the relative position of the object relative to the camera rig. RANSAC [20] was used in order to detect and reject outliers. Consequently, approximately 4% of the estimations were considered outliers and were discarded. The parameter values used in this numerical estimation are summarized in Table 1.

### 5.2. Results

**Figure 8:**
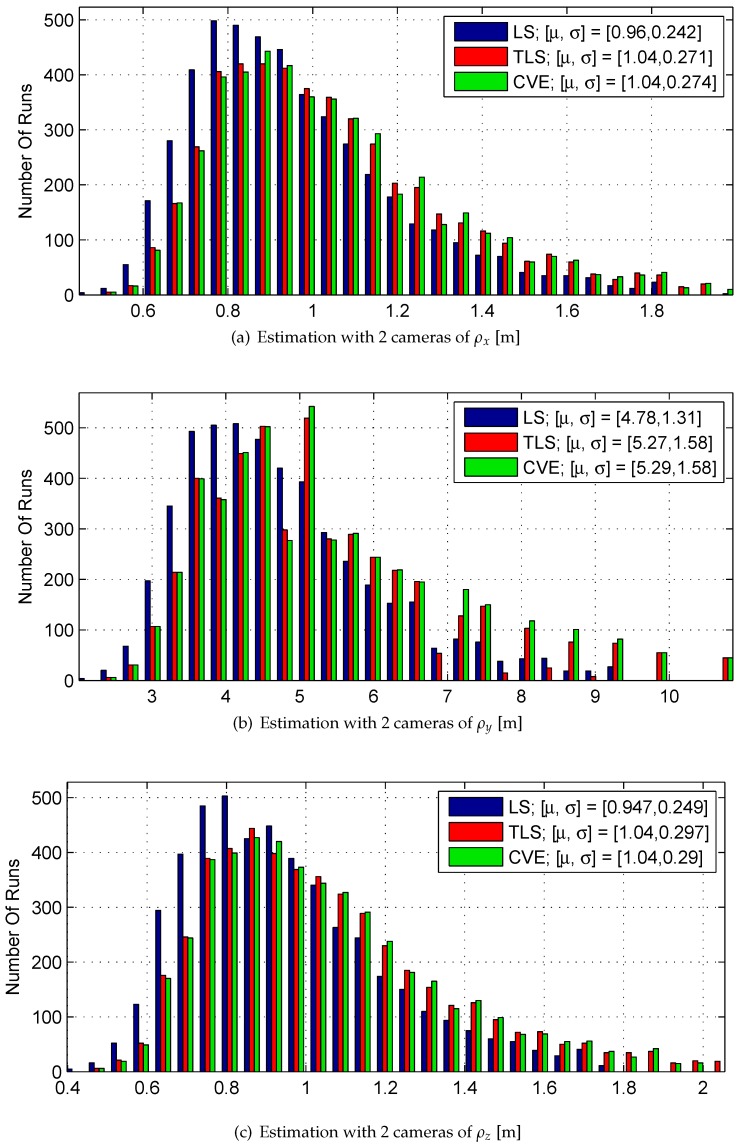
Monte carlo results of a static LOS estimation using stereovision measurements with 2 cameras.

**Figure 9:**
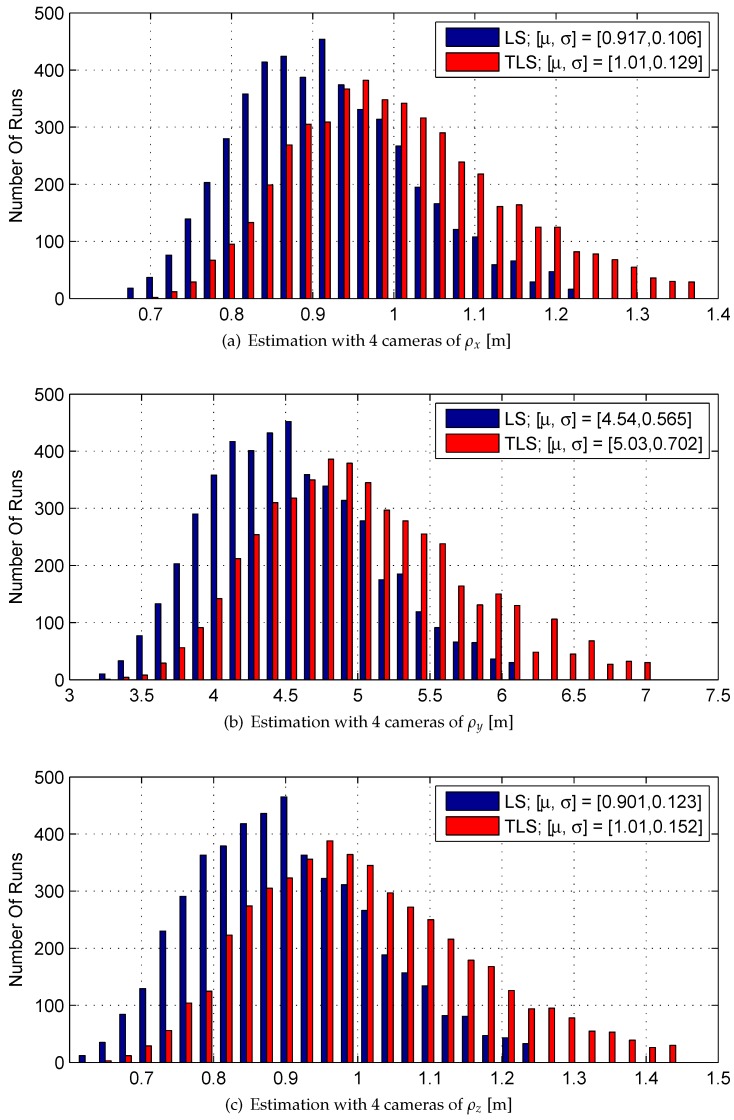
Monte carlo results of a static LOS estimation using stereovision measurements with 4 cameras.

Table 2 and Table 3 provide the statistical properties of the histograms depicted in Figure 8 and Figure 9, respectively. μ and σ denote the mean value and standard deviation, respectively. ${\left(\sigma_{LS}\right)}_{Est}$ is the estimation of the standard deviations of $\rho_{x}$, $\rho_{y}$, $\rho_{z}$ in the case of 2 cameras, which is estimated using Equation (18).

Although the estimation bias is not negligible with LS, TLS and CVE with 2 or 4 cameras, TLS and CVE produce significantly less bias than LS. Also, the use of 4 cameras does not always yield less bias compared to 2 cameras.

In the *y* direction, TLS and CVE produce slightly greater dispersion than LS. As expected, the use of 4 cameras yields less dispersion than 2 cameras. ${\left(\sigma_{LS}\right)}_{Estimation}$ is a fair approximation of $\sigma_{LS}$ because their corresponding components share the same magnitude.

According to these results, for the case of 2 cameras, LS, TLS, and CVE have similar performance. In this research TLS is used. For the case of 4 cameras, TLS is preferable over LS because of the bias improvement, while the dispersion is only slightly greater.

## 6. Experimental Results

The algorithms developed herein were tested in a series of experiments. These algorithms include image acquisition software, and control software, which achieve real-time performance. As mentioned in Section 1, the experimental part includes 2 cameras.

The sampling frequency of the computer-vision software is mainly dependent on the number of discovered feature points, and the size of the target projection in the frames. As a result, the computer-vision software’s time step varies between 0.4 and 0.6 s. The control software operates at a constant sampling frequency of 30 Hz.

The initial state covariance for all the filters is a 6×6 diagonal matrix, whose main diagonal is $\left[4\;m^{2},4\;m^{2},4\;m^{2},225\;m^{2}/s^{2},225\;m^{2}/s^{2},225\;m^{2}/s^{2}\right]$. The values for [$\sigma_{x},\sigma_{y},\sigma_{z}$] for the process noise covariance, as mentioned in Section 3.1, are [1,1,0] m/s${}^{2}$, [0.5,0.1,0] m/s${}^{2}$ and [0.8,0.8,0] m/s${}^{2}$ for the EKF, UKF, and for the CMKF, respectively. Because only 2 cameras are used, the measurement noise covariance for the EKF and UKF is a 4×4 diagonal matrix. Its main diagonal is $\left[25\;{\mathrm{px}}^{2},100\;{\mathrm{px}}^{2},25\;{\mathrm{px}}^{2},100\;{\mathrm{px}}^{2}\right]$ For the CMKF, the measurement noise covariance is calculated in each step, as described in Section 3.3. For that, ΔZ is assumed to be 10 pixels. Also, the UKF algorithm in this research uses a simple uniform set of weights [13].

### 6.1. Laboratory Description

**Figure 10:**
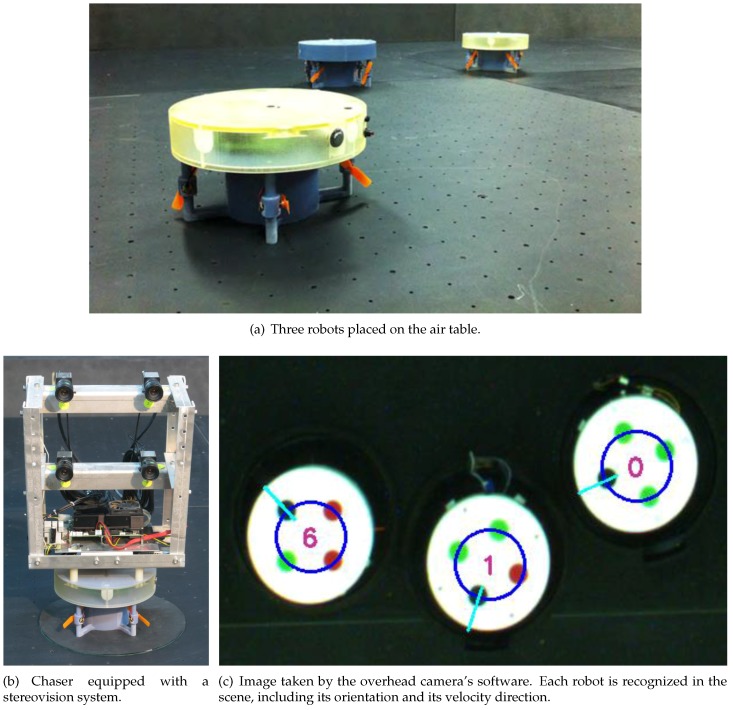
The equipment in the DSSL.

The experimental part of this research was conducted in the DSSL at the Technion. The DSSL is equipped with an air table, floating robots (Figure 10a), communication system, main computer, a stereovision camera rig (Figure 10b) and an overhead camera (Figure 10c), which measures the position and orientation of the robots on the air table.

Each robot is characterized by four circles with different colors in different order (Figure 10c). The overhead camera uses those colors to identify each robot and determine its orientation. The overhead camera is calibrated so as to relate the position of each robot in the image plane to its corresponding inertial position.

**Figure 11:**
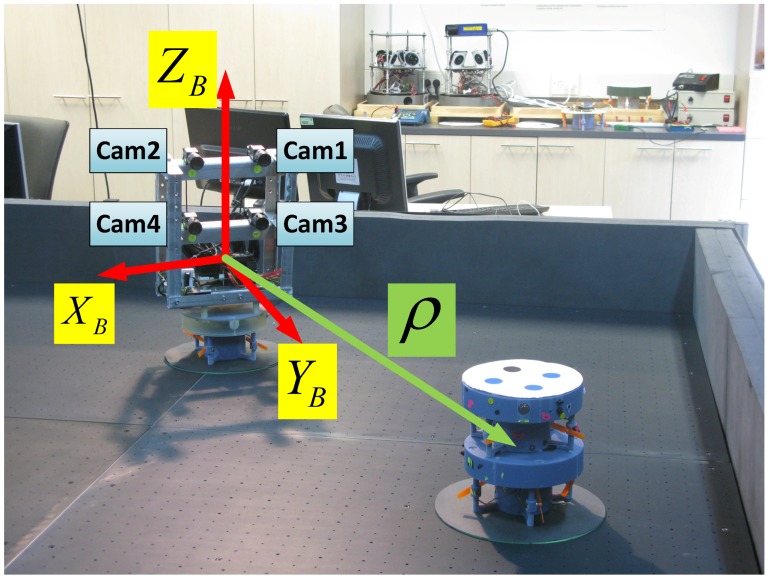
The body frame, camera numbers and LOS vector, shown in the laboratory environment.

Figure 11 depicts the chaser with the camera rig facing the target. As can be seen in Figure 11, the camera rig contains four cameras, which are all aligned. Notice that although the mounted camera rig does include four camera, the experimental part of the research utilizes only two of them (the top two, which are marked as “Cam1” and “Cam2” in Figure 11).

### 6.2. Reference Signals

In the experiments, the values $\rho_{x}^{C}$, $\rho_{y}^{C}$, ϕ, $\theta_{c}$ are estimated and compared to reference values, where the corresponding reference values are calculated in the following manner. The overhead camera (see Section 6.1) acquires measurements of the position of the chaser and the target in the inertial frame ${\vec{x}}_{c}^{I}$, ${\vec{x}}_{t}^{I}$, and the orientation of the chaser $\theta_{c}$. $\rho_{x}^{C}$ and $\rho_{y}^{C}$ are calculated using the equation

(46) $${\vec{\rho }}^{C}=\left[\begin{array}{c} \rho_{x}^{C}\\ \rho_{y}^{C} \end{array}\right]=\left[\begin{array}{cc} \cos\theta & \sin\theta \\ -\sin\theta & \cos\theta \end{array}\right]{\vec{\rho }}^{I}$$

The LOS azimuth angle ϕ is calculated using Equation (41).

### 6.3. FOV Properties

The parameter values used in this research are given in Table 4. Using Equations (37), (38), (40) and Table 4, StereoView and $Range_{min}$ are calculated,

(47) StereoView=0.73Range−0.16[m]

(48) $$Range_{min}=0.218\;m$$

(49) $$\phi_{max}\left(Range\right)=\arctan\left(0.367-\frac{0.08\;[m]}{Range}\right)$$

### 6.4. Static Experiment

**Figure 12:**
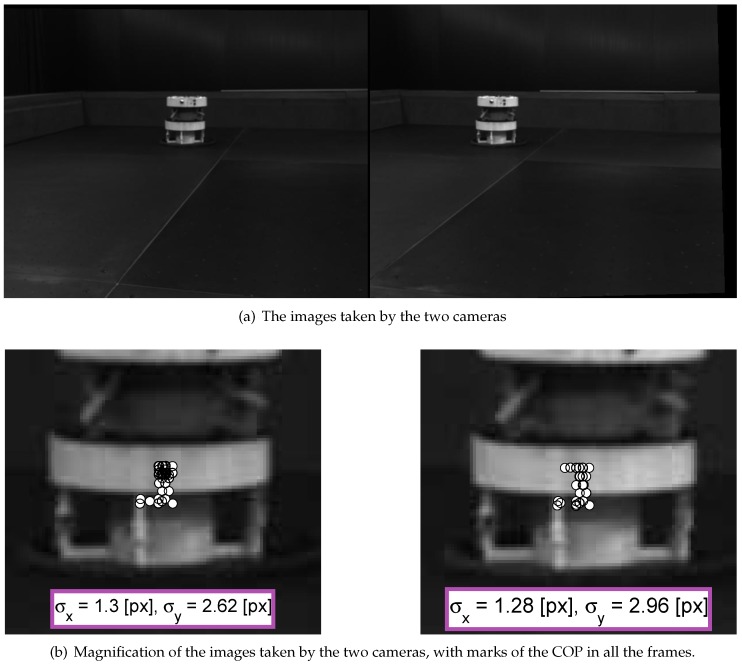
The images taken by the two cameras, with a statistical analysis of the COP dispersion.

A static experiment was conducted, wherein the target and chaser are placed at fixed locations. The purpose of this experiment is to test the target recognition algorithm. Due to measurement noise, which is manifested by a different number of feature points and locations, the target’s COP in each frame is slightly different, as seen in Figure 12. It is desired to measure this dispersion in order to use it in the estimation algorithm.

**Figure 13:**
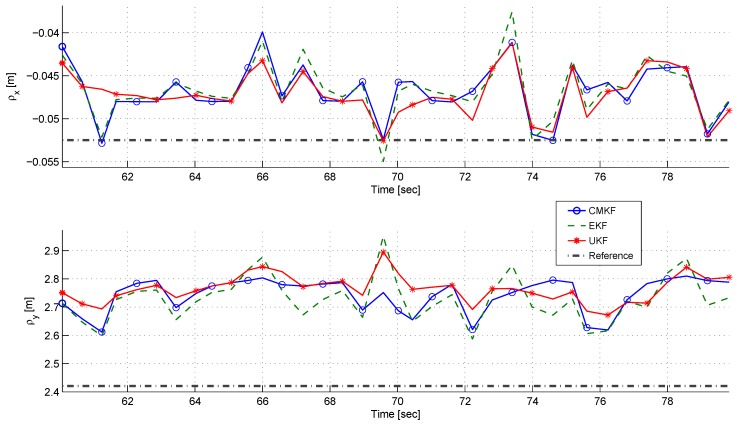
A comparison of $\rho_{x}$ (**top**) and $\rho_{y}$ (**bottom**) estimation using different filters in the static experiment.

In this experiment, due to the prominence of the color of the target’s bottom compared to its top, a larger number of features was found at the bottom. Consequently, the COP location was estimated to be lower than expected. As seen in Equation (15), this has minor to no effect on the components of the LOS estimation in the *x* and *y* directions. The main influence on these components is the COP’s horizontal position. As noted in Figure 13, the dispersion of the target’s COP is

(50) $$\sigma_{x}\approx 1.3\;\mathrm{px}\;\;\sigma_{y}\approx 2.8\;\mathrm{px}$$

Although the dispersions in both directions share the same magnitude, it is reasonable to assume that $\sigma_{y}$ is greater than $\sigma_{x}$ due to the target’s shape, which is characterized by a slightly longer vertical dimension.

**Figure 14:**
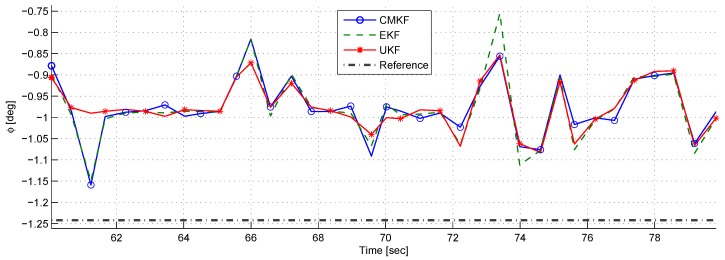
A comparison of the azimuth estimation using different filters in the static experiment.

Figure 13 and Figure 14 depict the LOS horizontal components and the azimuth estimation, respectively. It can be seen that the estimation of $\rho_{x}$ and $\rho_{y}$, and consequently ϕ, are biased, and that all of the estimated values are constantly over-estimated; that is, the estimated values are larger than the true values. The bias values are approximately

(51) $$bias\left(\rho_{x}\right)\approx 0.5\;\mathrm{cm}\;bias\left(\rho_{y}\right)\approx 30\;\mathrm{cm}\;bias\left(\phi \right)\approx 0.25\;\deg$$

It can also be seen that in the static estimation scenario, due to the slight changes in the target’s COP in each time step, the estimates of the filters do not converge, but stay around the average value. By using these results, it is not definite which estimator works best. The use of the CMKF did not produce better results than the EKF and UKF.

### 6.5. Semi-Static Experiment

A semi-static experiment was conducted where the target is stationary and the chaser had to maintain its position and orientation in order to keep the target within its FOV, while dealing with dynamic modeling inaccuracies and external disturbances.

Three impulses of external torques were applied during this experiment. These torques are marked in the different figures.

**Figure 15:**
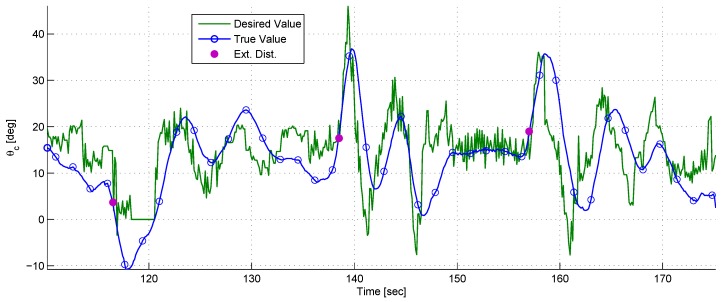
A comparison between the true body angle and the desired body angle.

Figure 15 depicts the chaser’s body angle $\theta_{c}$. It can be seen that after each impulse of external torque, $\theta_{c}$ converges to its nominal value with a characteristic response of a second-order system with two complex stable poles. This is expected, based on the rotational closed-loop dynamics modeled in Section 4.2.

Between 116 and 120 s, the target left the chaser’s FOV. As a result, $\theta_{c}$’s desired value is zero. While the chaser rotated towards $\theta_{c}=0$, the target returned to the FOV, the target recognition algorithm detected it and tracking was resumed.

**Figure 16:**
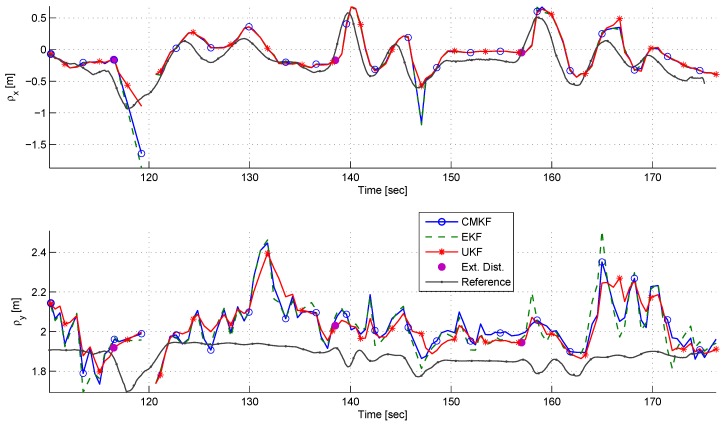
A comparison of $\rho_{x}$ (**top**) and $\rho_{y}$ (**bottom**) estimation using different filters in the semi-static experiment.

Figure 16 depicts the LOS components estimation as well as the reference values, where the reference values calculation is described in Section 6.2. The most prominent property in these graphs is the bias in the $\rho_{y}$ estimation. Most of the time this bias has a constant mean value with oscillations and occasional peaks. There is also bias in the $\rho_{x}$ estimation, but unlike the $\rho_{y}$ bias, the $\rho_{x}$ bias has negligible oscillations. Also, a good correspondence between the $\rho_{x}$ estimation and its reference is notable.

The absence of the target in the chaser’s FOV is seen in approximately 116–120 s and shortly after this event, the estimators diverge. In these sorts of events, the control algorithm is programmed to ignore the unreliable current estimate, and calculate the control signals according to a nominal predefined state. This divergence event ended when the target entered the FOV and tracking was resumed.

**Figure 17:**
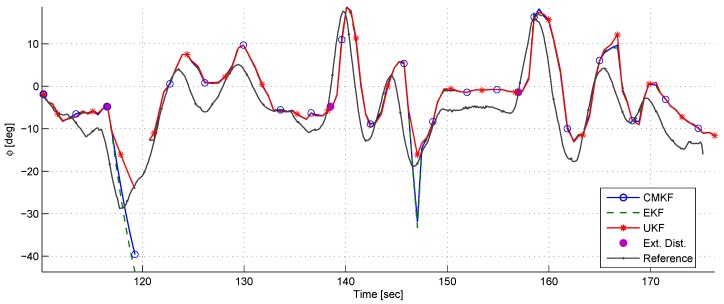
A comparison of the azimuth estimates using different filters in the semi-static experiment.

Figure 17 depicts the azimuth angle ϕ and its reference. ϕ is calculated using $\rho_{x}$ and $\rho_{y}$, and, therefore, all the different phenomena that occurred in $\rho_{x}$ and $\rho_{y}$ are reappearing in ϕ as well. It is also notable that the ϕ estimation is characterized by bias and delay.

At t=146, it can be seen that the CMKF and the EKF estimates had a large error compared to the UKF estimate. It is important to note that in this experiment the control signals were calculated using the UKF output.

**Figure 18:**
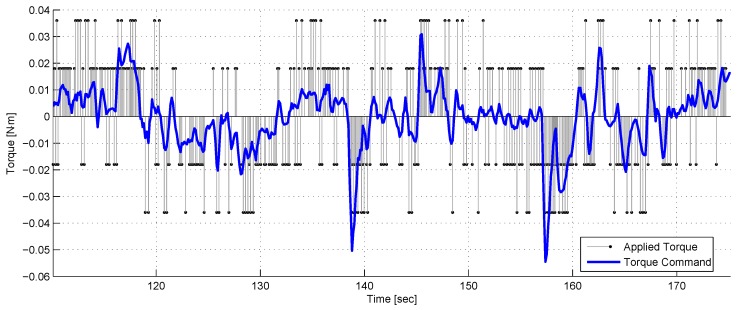
A comparison between the continuous torque command and the discrete applied torque.

Figure 18 depicts the torque commands versus the applied torque. As seen, the thrusters provide a discrete control torque although the commanded value is continuous.

### 6.6. Dynamical Experiment

A dynamical experiment was conducted, wherein the target moved in a cyclic trajectory. The chaser’s objective was to maintain its position and change its orientation in order to keep the target within its FOV.

**Figure 19:**
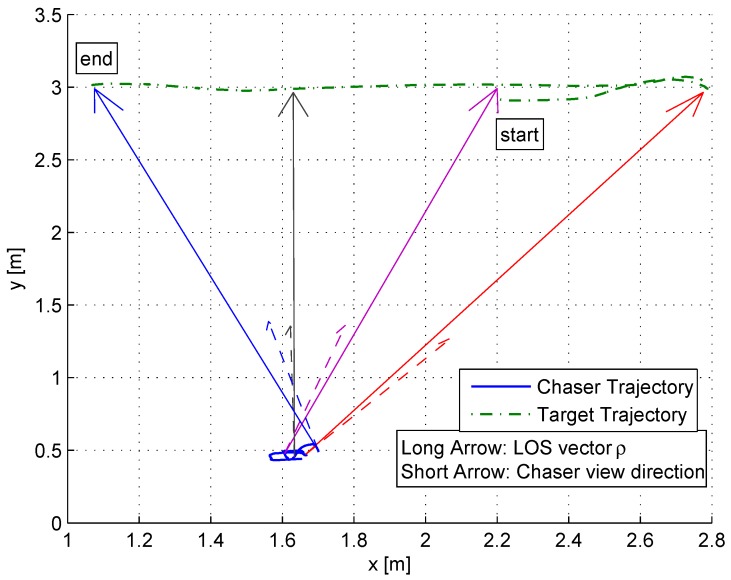
The trajectory of the target and the chaser including the LOS and body directions at different time steps.

Figure 19 depicts the trajectories of the chaser and the target during the experiment. In order to better illustrate the experiment, four points in time were selected. For each, the position and orientation of the chaser and the position of the target were marked.

The long arrows are the LOS vectors in different time steps, and the short arrows are the directions of the chaser’s body in each time step.

**Figure 20:**
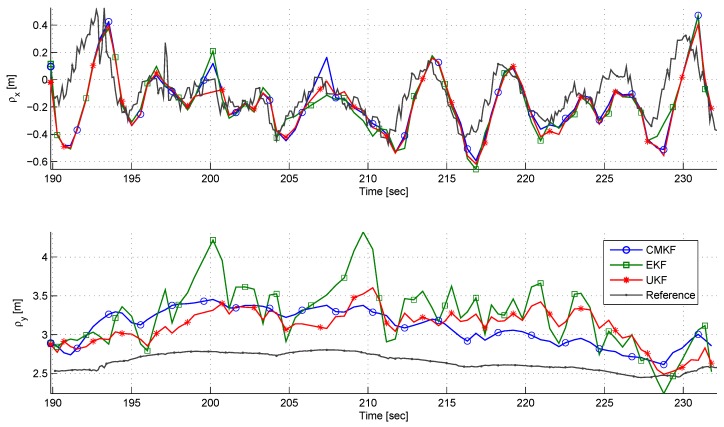
A comparison of $\rho_{x}$ (**top**) and $\rho_{y}$ (**bottom**) estimation using different filters in the dynamic experiment.

Figure 20 depicts the LOS components estimation and references. A bias is seen in the $\rho_{y}$ estimation. It is clearly seen that the EKF has the worst performance. On the other hand, the $\rho_{x}$ estimation has good correspondence to its reference with all estimators.

**Figure 21:**
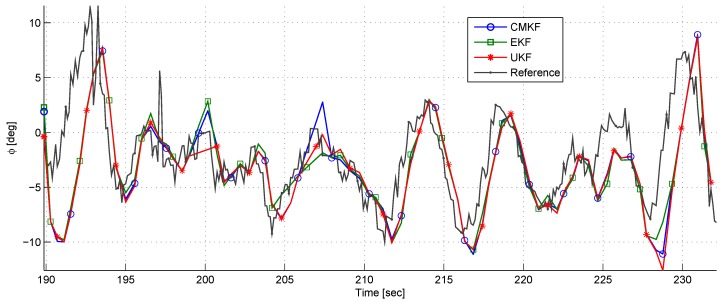
A comparison of the azimuth estimates using different filters in the dynamic experiment.

Figure 21 depicts the azimuth angle ϕ during the experiment. Although $\rho_{y}$ was constantly over-estimated, it can be seen that the azimuth angle has a good correspondence to its reference without bias.

During approximately 190–194 s and 227–231 s, a large error is seen in the azimuth angle. These large errors had likely occurred due to rapid and substantial changes in the relative position. It is important to remember that the estimation algorithm has an approximately half a second delay.

**Figure 22:**
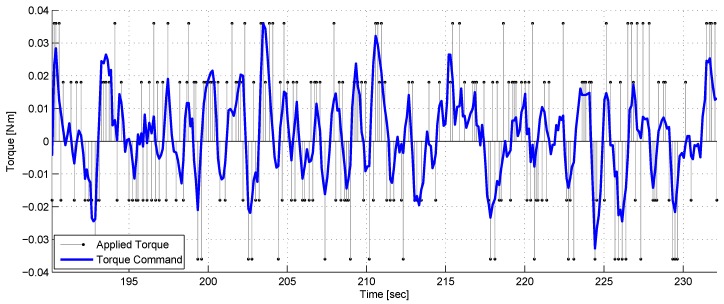
A comparison between the continuous control torque signal, the discrete applied torque and the PD signal.

Figure 22 depicts the torque commands versus the applied torque. As seen, the control signal oscillates, which implies that the dynamical system does not reach an equilibrium. This happens because as long as the target continues moving, the chaser has to keep adjusting its orientation.

### 6.7. Error Analysis

The results in Section 6.4, Section 6.5 and Section 6.6 exhibit a bias in the LOS depth component’s estimation. It seems that for stereovision with two cameras, bias is an inherent property of the system. This bias was also seen in Figure 8 and in previous work [10].

An interesting issue worth discussing is the fact that although the LOS depth component $\rho_{y}$ is biased, the estimated azimuth angle ϕ is quite accurate. Recall that

(52) $$\phi =\tan^{-1}\left(\frac{\rho_{x}}{\rho_{y}}\right)\approx \frac{\rho_{x}}{\rho_{y}}$$

and hence

(53) $$\Delta \phi \approx \sqrt{{\left(\frac{\partial \phi }{\partial \rho_{x}}\Delta \rho_{x}\right)}^{2}+{\left(\frac{\partial \phi }{\partial \rho_{y}}\Delta \rho_{y}\right)}^{2}}\approx \frac{1}{\rho_{y}}\Delta \rho_{x}+\frac{|\rho_{x}|}{\rho_{y}^{2}}\Delta \rho_{y}\;\left(\rho_{y}>0\right)$$

Equation (53) shows that the errors $\Delta \rho_{x}$, $\Delta \rho_{y}$ contribute to the error Δϕ, but they are mitigated by $\rho_{y}$ and $\rho_{y}^{2}$, respectively. Since $\rho_{y}$ has a relatively large value, it reduces the error effect dramatically.

## 7. Conclusions

In this research, a stereovision based algorithm for detection of non-cooperative targets and estimation of the relative position in real-time was developed. The detection algorithm does not require any a priori information except the assumption that the target is the most dominant object in the image. Moreover, a real-time tracking algorithm, which utilizes the stereovision information and keeps the target within the FOV, was developed.

A numerical study, which studies solution methods for the measurement equations was performed, and experiments which utilized the different algorithms were conducted. In these experiments, the performance of three estimators was compared using experimental data.

Real-time performance of the estimation and LOS control algorithms was demonstrated in the experiments carried at in the DSSL. The experimental and numerical results show that there is a non-negligible bias in the LOS depth component estimation with all of the estimators. On the other hand, the LOS horizontal component is estimated with a much smaller bias. The semi-static experiment exhibited the target detection algorithm performance in case where the target leaves and returns to the FOV.

Although the three estimators have similar behaviour, it seems that the EKF has the poorest performance. Deciding which estimator is better, the UKF or the CMKF, is not trivial, because most of the time their performance is similar. On the other hand, in the semi-static experiment, it is shown that unlike the CMKF, the UKF manages to cope with rapid and substantial changes in the dynamics, and therefore it outperforms the CMKF.

By using SURF feature points, simplifying the detection algorithm and using a small state vector, the average time step duration is reduced compared to previous work to approximately 0.4 s, which for most applications, is approximately the upper bound for real time operation.

## Figures and Tables

**Table 1:** Simulation parameter values.

Parameter	Value	Units
$f=f_{eff}$	750	px
$\rho_{x}$	1	m
$\rho_{y}$	5	m
$\rho_{z}$	1	m
b=baseline	0.16	m
ΔZ	5	px

**Table 2:** Monte-Carlo results summary, 2 cameras.

Parameter	True Value	$\mu_{\mathrm{LS}}$	$\mu_{\mathrm{TLS}}$	$\mu_{\mathrm{CVE}}$	$\sigma_{\mathrm{LS}}$	${\left(\sigma_{\mathrm{LS}}\right)}_{\mathrm{Est}}$	$\sigma_{\mathrm{TLS}}$	$\sigma_{\mathrm{CVE}}$
$\rho_{x}$ [m]	1	0.96	1.04	1.04	0.242	0.272	0.271	0.274
$\rho_{y}$ [m]	5	4.78	5.27	5.29	1.31	1.473	1.58	1.58
$\rho_{z}$ [m]	1	0.947	1.04	1.04	0.249	0.295	0.297	0.29

**Table 3:** Monte-Carlo results summary, 4 cameras.

Parameter	True Value	$\mu_{\mathrm{LS}}$	$\mu_{\mathrm{TLS}}$	$\sigma_{\mathrm{LS}}$	$\sigma_{\mathrm{TLS}}$
$\rho_{x}$ [m]	1	0.917	1.01	0.106	0.129
$\rho_{y}$ [m]	5	4.54	5.03	0.565	0.702
$\rho_{z}$ [m]	1	0.901	1.01	0.123	0.152

**Table 4:** FOV Properties.

Parameter	Value	Units
SensorWidth	6.784	mm
$f_{base}$	8	mm
$R_{F}$	0.5	
α	40.3	deg
baseline	0.16	m
